# A Review of Registered Clinical Trials on Dietary (Poly)Phenols: Past Efforts and Possible Future Directions

**DOI:** 10.3390/foods9111606

**Published:** 2020-11-04

**Authors:** Mirko Marino, Cristian Del Bo’, Daniela Martini, Marisa Porrini, Patrizia Riso

**Affiliations:** 1 Department of Food, Environmental and Nutritional Sciences (DeFENS), Università degli Studi di Milano, 20133 Milan, Italy; mirko.marino@unimi.it (M.M.); cristian.delbo@unimi.it (C.D.B.); marisa.porrini@unimi.it (M.P.); patrizia.riso@unimi.it (P.R.); 2 CRC “Innovation for Well-Being and Environment (I-WE)”, Università degli Studi di Milano, 20122 Milan, Italy

**Keywords:** bioactive compounds, clinical trials, human nutrition, health outcomes, food, food extracts

## Abstract

In recent years, the increasing number of studies on polyphenol demonstrates the efforts in elucidating the potential role of these bioactives on human health. This study reviews the main topics and characteristics of clinical trials on polyphenols registered over the last 20 years, in order to track past and current efforts as well as to highlight the main research gaps in this field. The review was conducted by collecting trials registered in ClinicalTrials.gov and International Standard Randomised Controlled Trial Number (ISRCTN) registry. Overall, 750 clinical trials were selected and included in the final evaluation. Most of the trials were performed on extracts or pure compounds followed by studies conducted on polyphenol-rich foods, in particular berries. A total of 520 clinical trials focused on health effects, 55 on bioavailability, and 175 on both. Regarding outcomes, 139 registered intervention studies had the lipid profile and blood pressure as primary outcomes. The overview provided by this analysis also emphasizes the emerging interest in new outcomes related to polyphenols intervention such as microbiota composition and the evaluation of inter-individual variability in response to the intake of polyphenols. Our review underlines the need of further trials covering unexplored or debated research aspects and provides insights for the design and development of future intervention studies and related research areas.

## 1. Introduction

The role of dietary bioactives has gained growing importance in food and nutrition research, as demonstrated by the increase in clinical trials. Among the different classes of dietary bioactives, polyphenols have been the focus of a large number of publications over the past 20 years. For example, the published literature retrieved in PubMed with the search term “polyphenols” grew from 1383 to 9600 publications from 2000 to 2010, and in March 2020 had reached around 36,000 publications [1].

This huge increase in polyphenol research is probably firstly due to the fact that they are a large family of hundreds of secondary metabolites with a diverse structure that can be found in many different edible plants [2,3,4,5] and that are consumed daily through different food products and preparations. Secondly, there has been a substantial evolution in food and nutritional sciences observed over the past 20 years. In fact, the concept of optimal nutrition, aimed at optimizing body functions and promoting human health, has to a large extent replaced conventional nutritional recommendations (i.e., focused on ensuring the amount of nutrients required for growth, to avoid nutritional deficiencies and related diseases) [6].

Bioactive compounds, other than macro- and micronutrients, seem to play an important role in the context of optimal nutrition and thus need further investigation. Although these compounds are not essential, they may modulate biological and physiological functions that promote health by reducing those factors known to increase the risk of age-related chronic diseases. In fact, there is a great demand for means to promote healthy aging [7]. This is due to the rise in the mean age of the global population plus the increase in the most chronic-degenerative diseases, also known as noncommunicable diseases, such as cardiovascular diseases, cancers, respiratory diseases, and diabetes. In this regard, several studies reported the capacity of polyphenols and polyphenol-rich foods to exert antinflammatory, antioxidant and vasoactive properties [8]. In addition, polyphenols have been documented to positively modulate glucose response, blood pressure and lipid profile, thus contributing to an overall improvement of cardiometabolic health [8,9,10]. Furthermore, polyphenols have been recently studied for their potential role in positively influencing the gut microbiota composition [11,12,13].

Despite the high number of studies on polyphenols, current evidence is mostly based on findings from observational studies [14,15,16], as well as from in vitro studies which have revealed the potential mechanisms through which polyphenols may exert their protective effects. However, in order to clarify the effects of polyphenols on specific body functions and biological activities there is an increasing demand for clinical trials performed on target populations [17]. These studies should also consider variability of the polyphenol content of foods, as well as the high inter-individual variability (e.g., in terms of bioavailability), which might explain why some individuals benefit more than others from the intake of these bioactives [18]. Evidence from human trials can be key to providing further insights aimed at establishing dietary reference intakes for these compounds. This is because, based on current data, it is still difficult to establish an evidence-based reference intake for the whole class and all the subclasses of these compounds, and thus well-designed and methodologically sound research in this field is needed [19]. Past and current research efforts on the topic can thus be used to analyze the trends over time together with the priorities identified to date. We therefore performed a review on polyphenol research of all the registered clinical trials in the last 20 years, by retrieving the information on registries of clinical trials. The aim was to highlight the main goals of the studies and their characteristics in terms of experimental design and outcomes, also providing insights into the main research gaps in this field.

## 2. Materials and Methods

### 2.1. Database Search Strategy

Our review was conducted using “ClinicalTrials.gov” [20] and International Standard Randomised Controlled Trial Number (ISRCTN) registry [21]. The focus was on clinical trials investigating polyphenols and performed all around the world from 2000 to March 2020. The first search was conducted on 30 January 2020. Intervention studies registered in ClinicalTrials.gov and (ISRCTN). registry were searched again on 1 April 2020 to identify additional studies. The search strategies involved the combination of the following terms using a syntax that was adapted for each registry:

- ClinicalTrials.gov: polyphenols OR flavonoids OR flavanols OR anthocyanidins OR anthocyanins OR isoflavones OR flavones OR flavonols OR flavanones OR flavanonols OR nonflavonoids OR phenolic acids OR stilbenes OR lignans.
- ISRCTN registry: (“polyphenols”) OR (“flavonoids”) OR (“flavanols”) OR (“anthocyanidins”) OR (“anthocyanins”) OR (“isoflavones”) OR (“flavones”) OR (“flavonols”) OR (“flavanones”) OR (“flavanonols”) OR (“nonflavonoids”) OR (“phenolic acids”) OR (“stilbenes”) OR (“lignans”).

**Figure 1:**
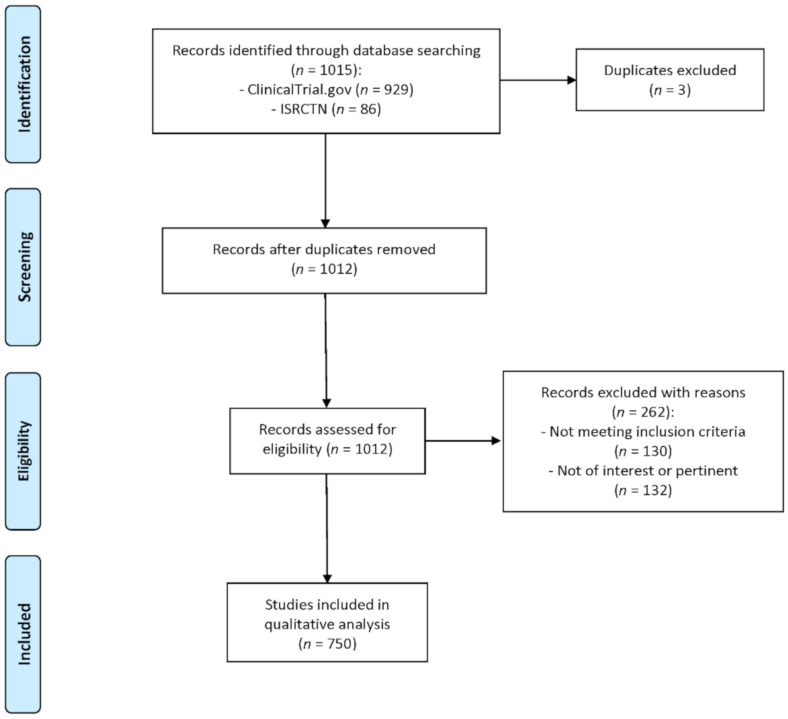
Flow diagram of the literature search process. Legend: ISRCTN: International Standard Randomised Controlled Trial Number.

The search strategy is summarized in Figure 1.

### 2.2. Study Selection

Studies were considered eligible if they consisted of human intervention studies investigating polyphenol bioavailability or the effects of polyphenols on human health. The search was limited to clinical trials registered between 2000 and March 2020. No restrictions on the characteristics of the participants were applied and the studies were included both if the interventions concerned polyphenol-rich foods and if polyphenols were provided as extracts and/or pure compounds.

The only exclusion criteria adopted was the use of polyphenols in combination with other nutrients or dietary bioactives or drugs, in order to select studies focused only on the effects of polyphenols. No other specific restrictions for the selection of the studies were applied.

Table 1 provides a more detailed list of eligibility criteria, developed by following the Population, Intervention, Comparison, Outcome, Study design (PICOS) format [22,23].

Two independent reviewers (M.M. and D.M.) conducted the study selection in the scientific databases and evaluated the eligibility of the clinical trials. Discrepancies between reviewers were solved through consultation with a third independent reviewer (C.D.B.) to achieve a consensus.

### 2.3. Data Extraction and Analysis

Data extraction from the registration of intervention studies in ClinicalTrials.gov and ISRCTN registry was performed by two reviewers (M.M. and D.M.). A third author (C.D.B.) checked the extracted information in order to ensure the accuracy of the data reported. For each study, the following information was collected: registration number, registration year, location, funding, participants’ information, study design, intervention, health condition, outcome measures. Studies were classified into four main categories based on their start date (2000–2004, 2005–2009, 2010–2014 and 2015–2020), similar to previous studies trends of several outcomes along the years [24,25,26]. Trials before 2000 were not considered while those started during the first three months of 2020 were included in the last category, from 2015 to 2020. Within these different time intervals, all studies were then further divided into two sections: polyphenol-rich foods and polyphenol-rich extracts or single pure compounds. Regarding the study location, countries were classified as “low” (number of registered studies in that country less than 10), “medium” (10 to 49) and “high” (50+).

## 3. Results

### 3.1. Study Selection

A total of 1015 registered clinical trials, conducted between January 2000 and March 2020, were identified from the database search (ClinicalTrials.gov and ISRCTN registries). After excluding 3 duplicates, 1012 records were assessed for eligibility. Out of these, 262 records were removed as they were not pertinent (*n* = 132) or because they did not match the inclusion criteria and specifically because polyphenols were provided in combination with other nutrients or bioactives (*n* = 97), or in combination with drugs (*n* = 33).

A total of 750 registered clinical trials were included in the final evaluation, as shown in Table 2. Out of these 750 clinical trials, 510 studies were chronic interventions, and 183 were acute. A total of 57 studies included both chronic and acute interventions.

Most of the studies were randomized, double-blind, placebo-controlled trials, especially if they were performed using extracts (about 80%) rather than whole polyphenol-rich foods (67%). The parallel-arm was the most common design for clinical trials on extracts (63% of the studies), and the cross-over design was the most common in intervention studies on polyphenol-rich foods (61% of the studies), with a similar trend over the years.

### 3.2. Trials on Polyphenol-Rich Foods and Extracts

**Figure 2:**
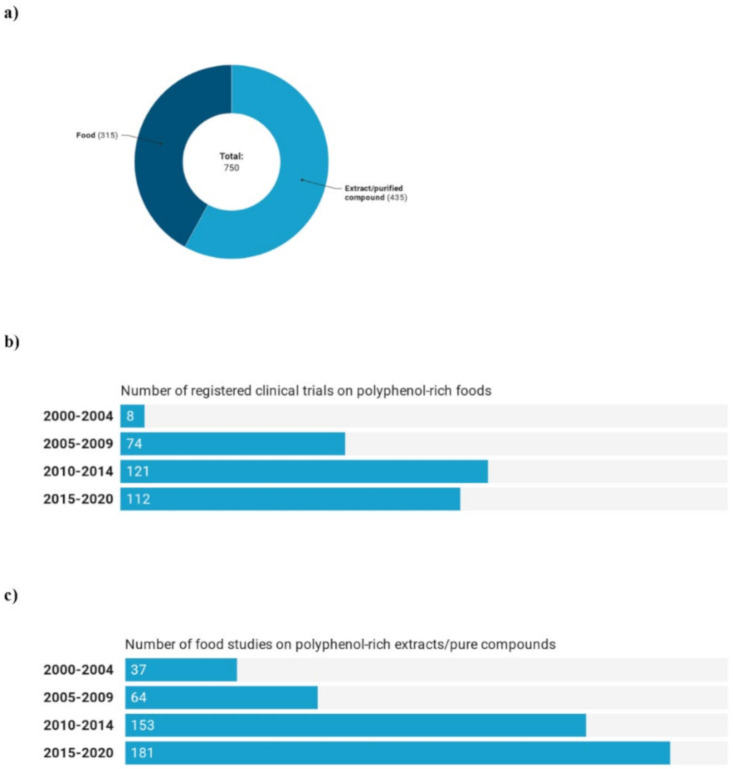
Number (**a**) and trend of registered trials on polyphenol-rich foods (**b**) and extracts or pure compounds (**c**).

Analyzing the whole period considered (2000–2020), 42% of clinical trials were performed on polyphenol-rich foods, and 58% were conducted on extracts or pure compounds (Figure 2a). Overall, considering the different time periods (Figure 2b,c), there were more studies on extracts or pure compounds than on polyphenol-rich foods, except for 2005–2009. Moreover, studies on extracts or pure compounds increased in the different time periods, while there were fewer studies on foods in the last period compared to those in the previous five years (2010–2014).

#### 3.2.1. Types of Polyphenol-Rich Foods

**Figure 3:**
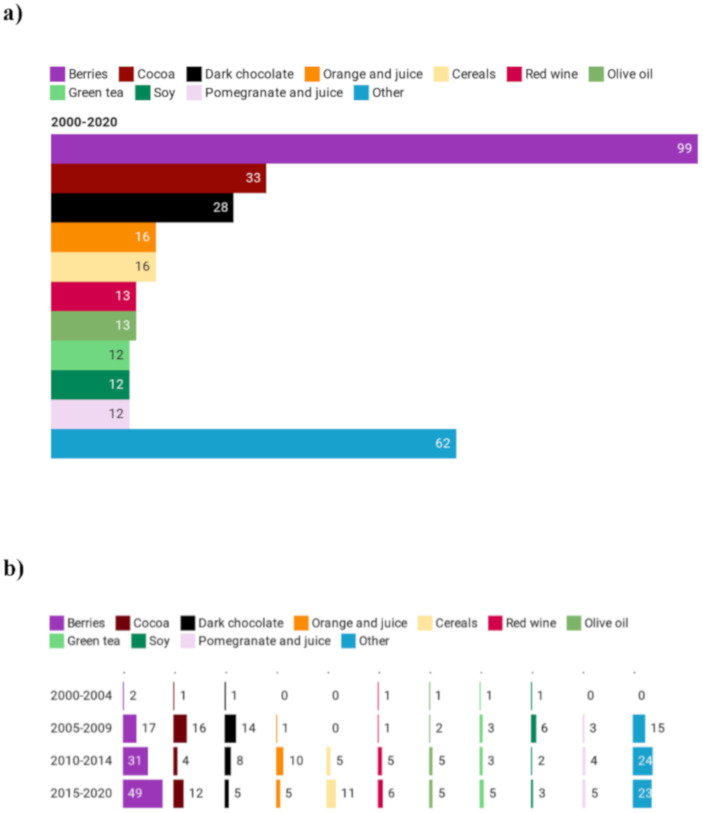
Main polyphenol-rich foods (**a**) used in clinical trials and their trend (**b**).

Berries were the most studied foods (Figure 3a), with a total of 99 registered trials, increasing (Figure 3b) up to 49 intervention studies from 2015 to 2020. Other frequently studied foods were cocoa and dark chocolate (33 and 29 clinical trials respectively, in the 20-year period considered) although from 2015 to 2020 fewer than 10% and 5%, respectively, of the 112 interventions focused on these products. Orange and orange juice, cereals, red wine, olive oil, green tea, soy and pomegranates accounted for a total of 94 studies, that is almost one third of all studies on polyphenol-rich foods. Finally, the category “other” included (i) generally less studied food sources (such as apples, coffee, potatoes, pulses and beer) and (ii) products were mainly considered in the last few years (such as hazelnuts, almonds, artichokes, mangos and dates).

#### 3.2.2. Types of Polyphenol-Rich Extracts

**Figure 4:**
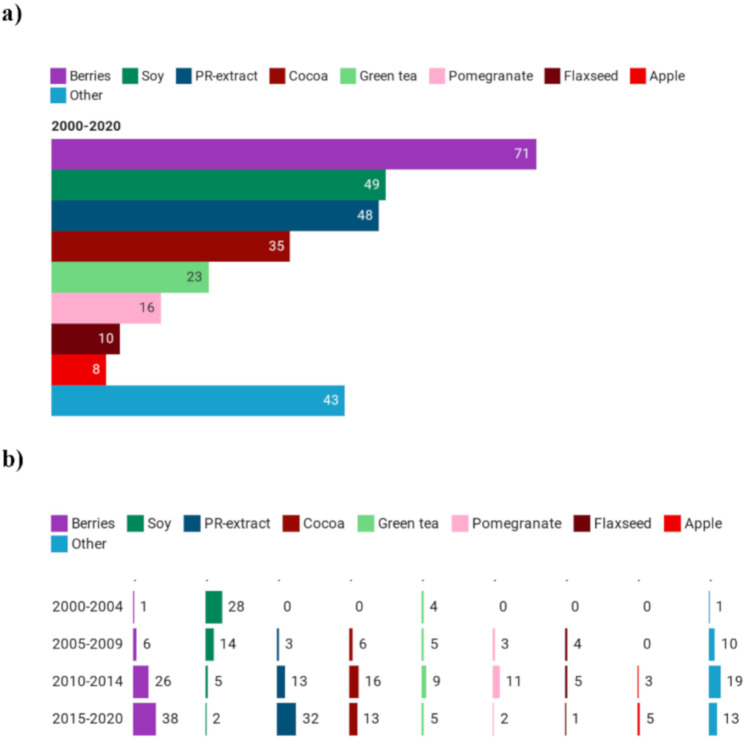
Main polyphenol-rich extracts (**a**) used in clinical trials and their trend (**b**).

Similarly to polyphenol-rich foods, the category of berries was the most studied (27% of the registered studies) (Figure 4a) also among intervention studies with extracts, with a constant increase over the years (Figure 4b). The second most studied category was soy extract whose interest, unlike berries, decreased over the years, from 28 trials between 2000 and 2004 to only 2 registered intervention studies from 2015 to 2020. Other food extracts studied included combinations of different food extracts (defined in Figure 4a as “PR-extract”, *n* = 48) and cocoa, green tea, pomegranates, flaxseeds and apple extracts which overall were considered in 92 studies.

#### 3.2.3. Types of Pure (Poly)Phenolic Compounds

**Figure 5:**
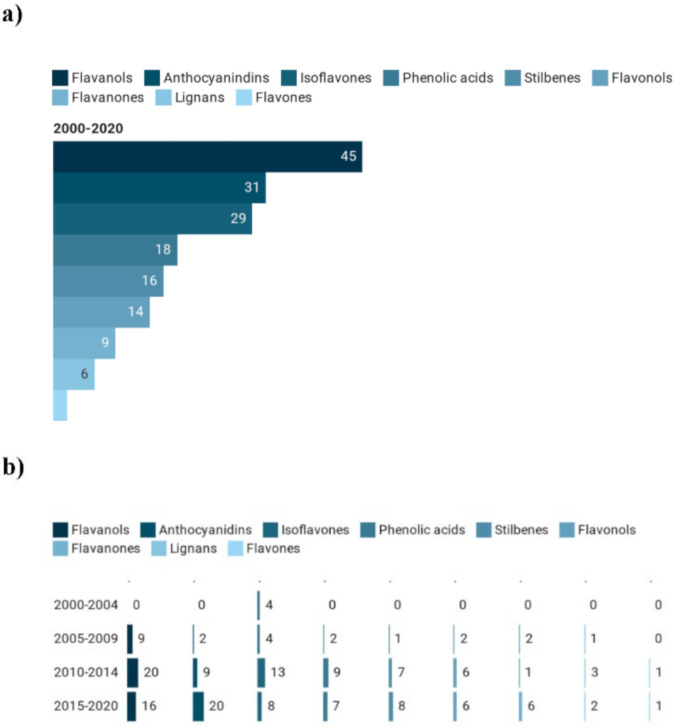
Main classes of pure polyphenols used in clinical trials (**a**) and their trend (**b**).

Figure 5a depicts the most considered classes of pure (poly)phenolic compounds used in registered clinical trials over the last 20 years. Flavanols were the most investigated class of polyphenols (*n* = 45 studies), followed by anthocyanidins (*n* = 31) and isoflavones (*n* = 29). However, as shown in Figure 5b, their trend differed according to the number of studies for each of the four periods. For instance, the highest number of registered trials in the 2010–2014 period focused on flavanols and isoflavones and then decreased in the last five years. Conversely, there was a growing trend for anthocyanidins for which there were no registered studies from 2000 to 2004 and 20 interventions from 2015 to today (27% of total studies on pure compounds in the last five years), thus becoming the most studied class of polyphenols.

### 3.3. Characteristics of Subjects

**Figure 6:**
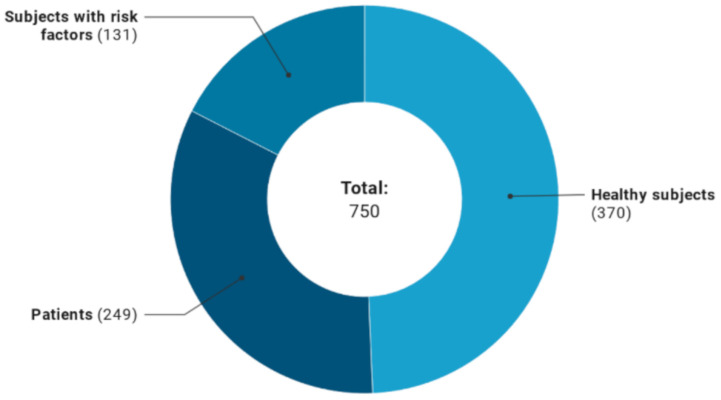
Health status of subjects included in the registered clinical trials.

Regarding the characteristics of the participants (Figure 6), most of the trials on polyphenols were conducted on healthy subjects (*n* = 370 studies, 49%), followed by studies on subjects with diseases such as cancer, urogenital diseases and mental disorders (*n* = 249, 33%), and then subjects with risk factors such as high blood pressure, fasting glucose and triglyceride levels (*n* = 131, 18%). Concerning age, 438 registered clinical trials included both adults and older subjects, 286 studies included only adults, 14 studies included only older individuals while 12 studies focused on children.

Registered clinical trials on polyphenols were balanced in terms of the participants’ sex, with 109 studies only on females and 114 studies only on males, while 527 studies included both sexes.

### 3.4. Main Goals of the Registered Trials

**Figure 7:**
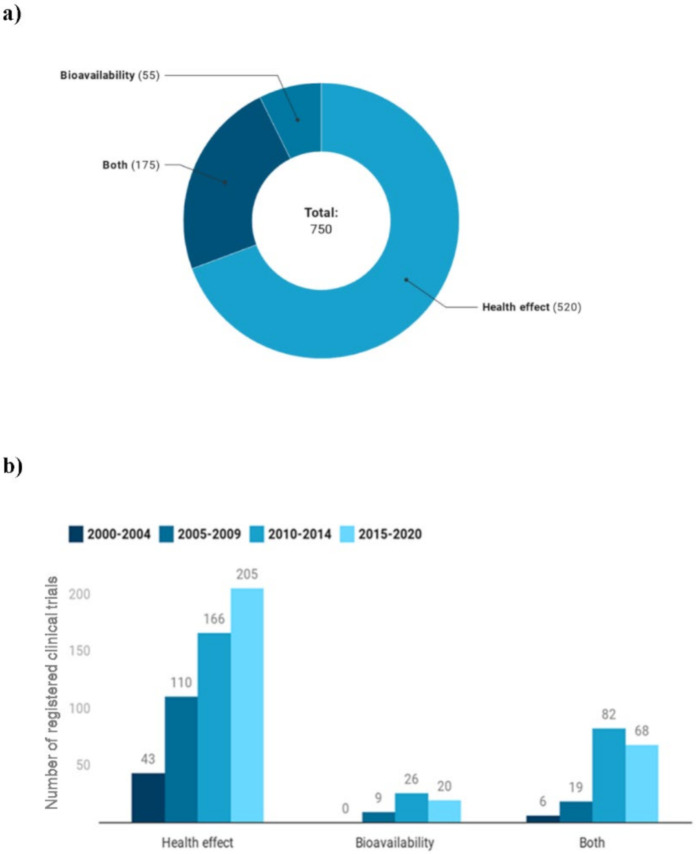
Number (**a**) and trend (**b**) of studies assessing the bioavailability or the health effects of polyphenols.

Figure 7a shows the main goals of the registered trials. A total of 520 clinical trials (197 on foods and 323 on extracts) focused on health effects, 55 (33 on polyphenol-rich foods and 22 on extracts) on bioavailability and 175 (85 on polyphenol-rich foods and 90 on extracts) evaluated both. Figure 7b highlights intervention studies on health effects increased over time, from 43 registered trials from 2000 to 2004, to 205 in the last five years. On the other hand, studies on bioavailability increased throughout the first three time periods (*n* = 0, 9 and 26, respectively) and then decreased in the 2015–2020 period (*n* = 20), similarly to studies assessing both health effects and bioavailability.

**Figure 8:**
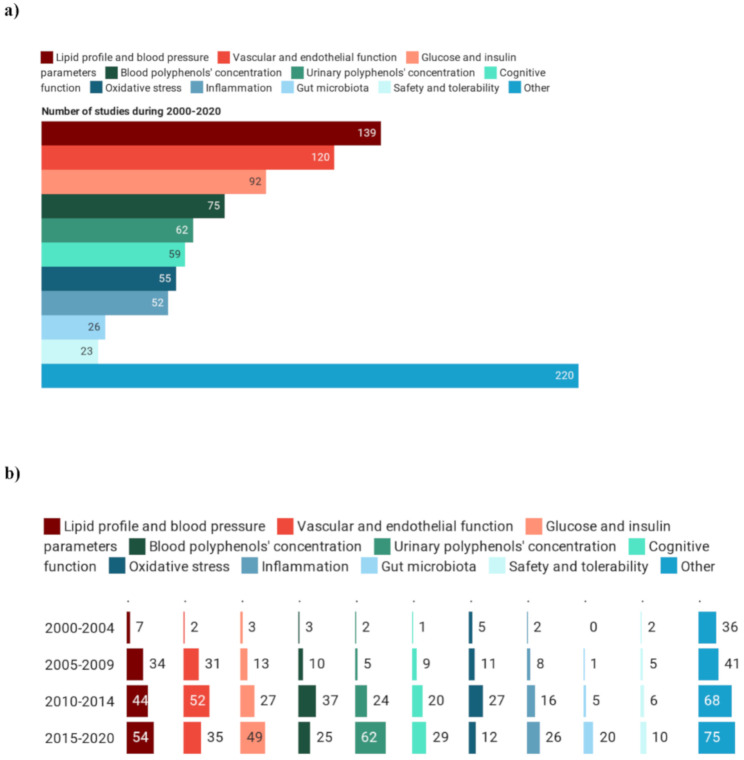
Primary outcomes (**a**) and their trend (**b**) assessed during clinical trials on polyphenols.

The outcomes of the registered trials are reported in Figure 8a. Lipid profile and blood pressure were the primary outcomes in 139 registered intervention studies, with a constant increase in absolute terms (from 7 in 2000–2004, to 34, 44 and 54 in the 2005–2009, 2010–2014, 2015–2020, respectively) but not in relative terms (e.g., 25% of the studies during the 2005–2009 period and 17% in the last five years) (Figure 8b). A similar trend was observed for studies on vascular and endothelial function, which had the highest number of interventions from 2005 to 2009.

In addition, glucose and insulin parameters were widely investigated, with a total of 92 intervention studies from 2000 to 2020. The number of studies on cancer and osteoporosis was high between 2000 and 2004 (23% and 21% of the total studies, respectively) and decreased drastically over time, accounting for 1% of the registered trials. Conversely, significant interest was recorded from 2015–2020 in primary outcomes that were neglected in the past, such as the modification in urinary polyphenol concentration and gut microbiota.

### 3.5. Other Characteristics of the Registered Trials

**Figure 9:**
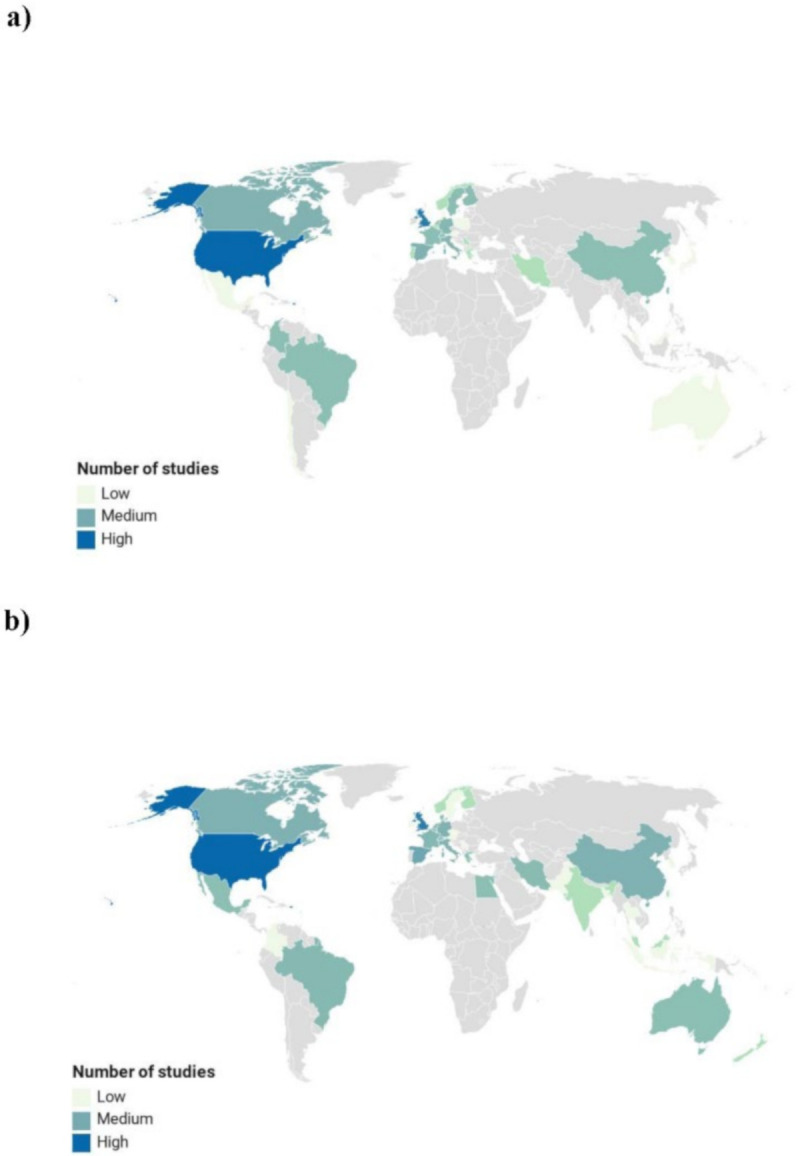
Countries with the highest number of registered studies on polyphenol-rich foods (**a**) and extracts (**b**). Legend: “low”: <10 registered trials; “medium”: 10–49 registered trials; “high”: >50 registered trials. Locations not reporting clinical trials on polyphenols are colored in grey.

Figure 9 reports the main countries where clinical trials on polyphenol-rich foods (Figure 9a) and extracts (Figure 9b) were registered. In each of the five-year periods considered, the highest number of registered studies was in the USA (230 clinical trials: 136 on extracts and 94 on polyphenol-rich foods) and in the UK (148 trials: 72 on extracts and 76 on foods). A significant number of interventions was also registered in Spain (*n* = 58), Canada (*n* = 43), Italy (*n* = 37), Germany (*n* = 34), China (*n* = 28), The Netherlands (*n* = 22), France (*n* = 20), Brazil (*n* = 17) and Switzerland (*n* = 15).

Other important information concerns the funding of the studies. A total of 580 and 170 registered clinical trials were supported by private and public funding, respectively, with a constant and increasing gap between the two types of funding. In fact, while public funding was prevalent from 2000 to 2004, the number of studies with private funding was threefold and fivefold those with public funding in the 2005–2010 and 2015–2020 periods, respectively.

## 4. Discussion

Human intervention studies are always in more demand due to the putative effects of food bioactives such as polyphenols. For the first time, we have documented the high number of human intervention studies on polyphenols currently registered in two of the main registries of clinical trials (i.e., 750 registered clinical trials included in the final evaluation), thus demonstrating the continuous and increasing interest in the role of these food bioactives. This is also supported by the high number of publications, together with the growing number of national and international research projects focused on polyphenols. Moreover, polyphenols represent an interesting and clear example of compounds that have attracted the attention of researchers from many different disciplines. First, there is a growing interest in the evaluation of the environmental, agronomic and pre-harvest aspects that can affect the polyphenol content in foods [27,28,29]. Secondly, food technology is making efforts to optimize traditional technologies and to develop novel techniques able to preserve the natural content of polyphenols in the raw materials as well as to use polyphenol-rich ingredients for food fortification [30,31,32,33,34]. Thirdly, experts in the nutrition and health related areas are committed to increase evidence in order to understand if and how it can be possible to develop dietary recommendations for polyphenols intake [9,19,35,36,37], also considering the demand for healthy and sustainable diets to be promoted as a potential climate change mitigation strategy. For these reasons, numerous different stakeholders, both in the private and public sector, seem to show a great interest for the potential applications of results from polyphenol research.

Regarding clinical trials, our findings show that chronic intervention studies tend to be more common than acute ones, above all in the last years. This implies a shift from studies mostly addressing polyphenol bioavailability to chronic trials that are more often focused on the evaluation of the medium and long-term effects of polyphenol intake on human health.

In view of the complexity of polyphenol pharmacokinetics and the increasing knowledge of the inter-individual variability in terms of absorption, distribution and metabolism affecting human responses to polyphenol intake [38,39,40], further studies are needed to better explore this variability and how this influences the impact of polyphenols on markers of human health [18,41,42,43]. These should also address the metabolic activities of microbiota. In fact, the importance of the microbiota-host interplay in comprehending the impact of polyphenols, and of diet in general, on human health is only recently emerging [39]. In detail, evidence suggests that polyphenols may act through modulation of the gut microbiota composition [44]. In turn, gut microbiota significantly affects the metabolic fate of plant polyphenols, as they undergo extensive metabolism leading to the release in the bloodstream of smaller and more polar metabolites which may be the responsible of several biological effects [12,45,46,47]. Taken together, these findings highlight the need to study the metabolism and health benefits together, rather than separately.

The increasing number of long-term and well-designed trials is an important achievement in terms of furthering our understanding of polyphenol intake (e.g., quantity and duration), which could lead to future recommendations regarding intake for the positive modulation of specific physiological functions [19]. Since these studies are costly, time-consuming, and often difficult to design and perform, their growing number suggests an increased investment in addressing the role of polyphenols in real settings and human subgroups of the population. In addition, it is noteworthy that studies on healthy or at-risk individuals prevail, in line with the guidance of the European Food Safety Authority reporting that subjects with a disease cannot be the target population for demonstrating a health claim made on food [48].

The high number of studies on the lipid profile, blood pressure, endothelial function and other related markers clearly highlights the wide interest in understanding the effects of polyphenols on cardiometabolic health. This is not surprising considering that, as highlighted by the Global Burden of Disease study [49], cardio-metabolic diseases are the leading cause of death worldwide, mainly triggered by the rise in biomarkers of metabolic syndrome, including waist circumference, fasting blood glucose, triglycerides, and blood pressure. Studies have underlined how a diet rich in plant-based foods, such as fruit and vegetables, could decrease the risk of these metabolic diseases, thanks to their content of fiber, vitamins, minerals but also bioactive compounds such as polyphenols [50,51] that can play an important role in the regulation of the redox status of cells. In addition they can improve endothelial function and vessel protection but also increase nitric oxide bioavailability, thus, playing a role on the maintenance of normal levels of blood pressure and on the inhibition of platelet aggregation [52,53].

In our review, there were less studies on the effects of polyphenol-rich foods than those investigating the effects of extracts or pure compounds, which are mostly used for investigating their bioavailability. This could be due to the increased interest in the industrial exploitation and development of food extracts for use as nutraceuticals. In details, among polyphenol-rich foods, there is still a great interest in berries due to their high content in phenolic compounds, such as phenolic acids, flavonols and anthocyanins and there are many papers demonstrating their positive role in the modulation of several physiological functions [54,55,56,57]. Conversely, a minor number of trials assessed the study of other polyphenol-rich foods on different outcomes, although evidence supports the positive role of other polyphenol-rich foods on markers of human health [58,59,60,61,62,63]. This may be due a lower interest and/or, more plausibly, to the lower availability of funding. In this regard, berries have also attracted private investments, probably being considered attractive and versatile foods for their exploitation in the pharmaceutical and food industry area. Funding could thus be increased in order to evaluate the effects of the combination of different foods and of polyphenol-rich diets, in order to provide insights into the role of these dietary models in a real-life setting.

We believe that a key strength of our study is that we opted to review registered trials rather than published papers in order to ensure a more up-to-date picture of the current research on a specific topic, also considering that papers are often published much later than the registration date and even the real end of the study. For instance, with this approach we were able to collect information of very recently registered trials, whose results will be likely published in the next few years. Moreover, within the registration form of a trial, the authors report some information that is sometimes not very clear in the manuscript (e.g., primary outcome, characteristics of subjects, funding). In fact, other reviews have analyzed registered clinical trials, for instance for investigating the characteristics of general registered trials [64], including sponsorship [65], or of specific topics such as ophthalmology [66], orthodontics [67], infection diseases [68], drug trials [69]. However, our review also has some limitations. First, the use of websites of trial registration does not allow to retrieve sufficient information to perform the risk of bias assessment, for instance because findings of the study are not included and thus, the bias of selection of the reported results and those of deviations from the intended intervention cannot be assessed [70]. Secondly, ClinicalTrials.gov and ISRCTN databases do not include all clinical trials as they are not the only databases available. Moreover, some trials may have not been included in the final evaluation, because they were not yet registered or because they did not use the keywords appropriately. An additional limitation could be that we excluded some clinical trials in which polyphenols were not the main bioactive compounds considered [71]. In this general context, it is emphasized the importance and relevance of the promotion and implementation of the practice of registering clinical trials for the analysis of trends and gaps in a research area.

## 5. Conclusions

Despite numerous data from literature have shown a protective role of polyphenols on human health, findings from the present review show that there is still a need of studies to fill several gaps in this field, also by considering the different factors such as individual response that could affect polyphenols bioavailability and bioactivity. In this scenario, databases such as ClinicalTrials.gov and ISRCTN. registry may play a key role to monitor registered intervention studies with the aim to track already ongoing research. The present work provides indeed useful insights to be used for the design of future studies in the field of polyphenols that will increase knowledge about the complex interaction between these compounds and the host in terms of bioavailability and biological response and their exploitation for the promotion of human health. The last but not the least, findings from such studies could provide a rationale and a driving force for activities aimed at improving nutritional profiles of promising products pushing towards the innovation in production, processing and the whole food system approach.

## Figures and Tables

**Table 1:** Population, Intervention, Comparison, Outcome, Study design (PICOS) criteria for trial selection cited.

PICOS Item	Inclusion Criteria
Population	Healthy or diseased children, adults and/or older adults
Intervention	Food, extract or pure polyphenols tested alone. No other bioactive compound or drug
Comparison	Control group without polyphenols
Outcome	Any effect on human health and bioavailability
Study design	No restriction on study design

**Table 2:** Characteristics of the included studies (*n* = 750).

	Foods (*n* = 315)	Extracts or Pure Compounds (*n* = 435)
*Goal*		
Health effect	197	323
Bioavailability	33	22
Both	85	90
*Duration*		
Acute	91	92
Chronic	194	316
Both	30	27
*Subjects*		
Healthy	174	196
Subject with risk factors	84	165
Patients	57	74
*Primary outcome*		
Lipid profile and blood pressure	74	65
Vascular and endothelial function	66	54
Glucose and insulin parameters	40	52
Blood polyphenols’ concentration	46	29
Urinary polyphenols’ concentration	36	26
Cognitive function	28	31
Oxidative stress	28	27
Inflammation	30	22
Gut Microbiota	18	8
Safety and tolerability	4	19
Other	98	122
*Location*		
USA	94	136
UK	76	72
Spain	23	35
Canada	21	22
Italy	17	20
Germany	13	21
China	4	24
Netherlands	4	18
France	6	14
Switzerland	10	5
Other	47	68
